# Retrobulbar Hematoma Following Orbital Floor Reconstruction

**Published:** 2013-04-30

**Authors:** Ingargiola Michael, Daniali Lily, J. Trovato Matthew

**Affiliations:** ^a^New Jersey Medical School—University of Medicine and Dentistry of New Jersey, Newark; ^b^Dallas Plastic Surgery Institute, Dallas, Tex

**Figure:**
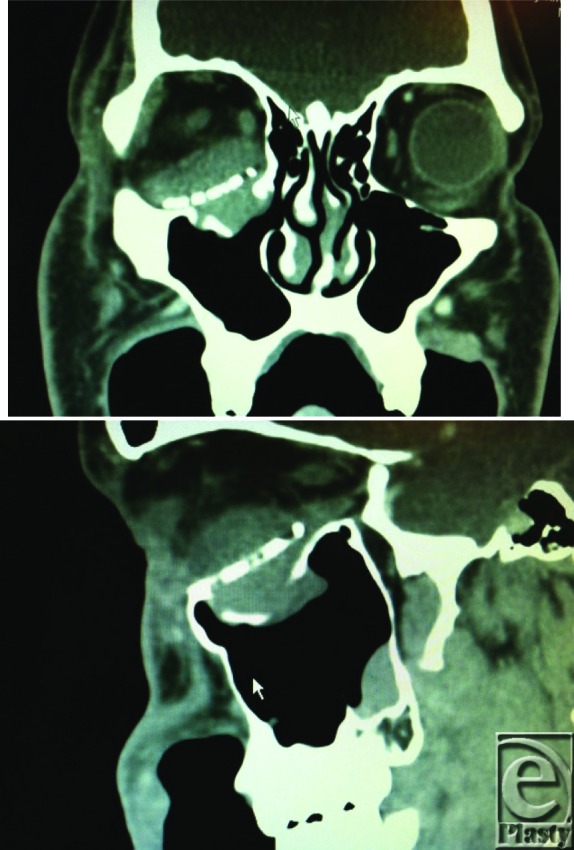


## DESCRIPTION

Ten days after reconstruction of an orbital floor blow out fracture with a porous polyethylene implant, a 23-year-old man presented with sudden onset of swelling and proptosis of the right eye following weightlifting.

## QUESTIONS

1. **What are the potential complications with reconstruction of orbital floor fractures?**
2. **What is the appropriate method of diagnosis of a retrobulbar hematoma?**
3. **What is the management of a retrobulbar hematoma?**
4. **What may have predisposed this patient to this complication?**

## DISCUSSION

The surgeon who endeavors to reconstruct the fractured orbital floor should be aware of the potential complications of surgical intervention. A recent retrospective review investigating the incidence of postoperative complications demonstrated 5.8% persistent motility impairment, 3.7% enophthalmos/hypothalamus, 3.2% diplopia, 2.6% ectropion, and 0.5% infection. The most severe reported complication was retrobulbar hematoma occurring in 3.2% of cases.^1^ Of patients who developed blindness following orbital surgery, 48% have been attributed to retrobulbar bleeding.^2^

Despite the well-documented incidence of retrobulbar hematoma, 83% of senior house officers in the emergency department were unable to diagnose and manage a retrobulbar hemorrhage appropriately in the acute setting.^3^ Retrobulbar hematomas occur primarily secondary to orbital trauma or surgery. The morbidity associated with delayed diagnosis or mismanagement is extremely high, and every physician should be familiar with the management algorithm to provide timely diagnosis and treatment to prevent permanent vision loss. Bleeding into an enclosed orbital space that cannot expand to accommodate the additional volume leads to an orbital compartment syndrome, with increased pressure exerted onto the optic nerve, retinal artery, and the vasculature of the optic nerve resulting in ischemia. Ischemia lasting 60 to 100 minutes can result in permanent visual loss.^4^^,^^5^

Rapid diagnosis and timely surgically decompression are the most crucial aspects of management of retrobulbar hematomas to prevent long-term visual impairment.^3^ In the conscious patient, clinical suspicion of a retrobulbar hematoma should be increased in a patient with proptosis complaining of decreased visual acuity or ocular pain with a mechanism of orbital trauma or surgery on history. Diagnosis in an unconscious patient may be more difficult, but suspicion should be increased with ocular proptosis and loss of direct pupillary reflex.^6^ Computed tomography is the gold standard for definitive diagnosis, and confirmation of increased ocular pressure can be made with tonometry. Chen et al evaluated the diagnostic work-up of retrobulbar hematomas and concluded that in the presence of diminished visual acuity, loss of light reflex, restricted extraocular movement, painful proptosis, and a stony eyeball, the diagnosis can be made clinically, allowing the clinician to proceed with treatment. When painful proptosis and a stony eyeball were not present, or the other reported symptoms were trivial, a CT scan was instrumental in clarifying the diagnosis.^7^

The cornerstone of treatment is timely orbital decompression followed by hematoma evacuation and control of any active bleeding. Lateral canthotomy and cantholysis is an accepted, effective, and quick intervention to decompress pressure off the orbit by increasing orbital volume.^8^ If immediate improvement is not seen, one must undergo emergent open exploration, particularly with the presence of a relative afferent pupillary defect. Medical interventions such as a megadose of corticosteroids, mannitol, acetazolamide, and topical timolol may mitigate the degree of ischemic injury and subsequent loss of vision and may be used as adjuncts to surgical decompression and hematoma evacuation.^9^^,^^10^

Retrobulbar hematomas typically occur acutely postoperatively, but they may occur up to a week after surgery.^11^^–^13 There are reported cases of retrobulbar hematomas occurring spontaneously after Valsalva with childbirth and vomiting in the migraine setting. A sudden increase in intrathoracic and abdominal pressure causes a subsequent increase in jugular venous pressure that is transmitted to the orbit by veins leading to a rupture with resulting hemorrhage and hematoma formation.^14^^–^16 The occurrence of a retrobulbar hematoma 10~days after surgery, as in this case, is extremely uncommon. The combination of weakness in the orbital structures following surgery coupled with a sudden increase in venous pressure due to Valsalva during weightlifting likely contributed to the development of his condition. Finally, the polyethylene implant may have impeded drainage of the fluid collection.
